# Pharmacovigilance insights: safety profiles of antifungal agents for invasive aspergillosis

**DOI:** 10.3389/fphar.2025.1718019

**Published:** 2026-01-07

**Authors:** Wei Jia, Tiezhou Wang, Jingru Wang

**Affiliations:** 1 Department of Pharmacy, Tonglu Branch Hospital, Hangzhou First People’s Hospital, Hangzhou, Zhejiang, China; 2 Department of Orthopaedics, Tonglu Branch Hospital, Hangzhou First People’s Hospital, Hangzhou, Zhejiang, China

**Keywords:** adverse event (AE), amphotericin B, caspofungin, FAERS, invasive aspergillosis (IA), isavuconazole, posaconazole, tailoring treatment

## Abstract

**Background:**

Invasive aspergillosis (IA) poses significant mortality risks, particularly in immunocompromised patients. The safety profiles of FDA-approved antifungal agents, triazoles (Voriconazole, Posaconazole, Isavuconazole), polyenes (Amphotericin B), and echinocandins (Caspofungin), are not yet fully characterized in real-world settings. This study employed pharmacovigilance data to systematically evaluate the comparative safety profiles of these agents, providing evidence-based insights for clinical practice.

**Methods:**

A retrospective analysis of the FDA Adverse Event Reporting System (FAERS) data (2004Q1–2024Q3) was conducted. Disproportionality analyses, including reporting odds ratio (ROR), proportional reporting ratio (PRR), Bayesian Confidence Propagation Neural Network (BCPNN), and Multi-item Gamma-Poisson Shrinker (MGPS), were employed to identify adverse event (AE) signals. Duplicate entries were identified and removed using CASE_ID and FDA_DT criteria, after which AE signals were classified according to MedDRA System Organ Classes (SOCs) and Preferred Terms (PTs).

**Results:**

Among 26,004 antifungal-associated AE reports, Amphotericin B exhibited the strongest renal toxicity signals (nephropathy toxic (i.e., nephrotoxicity): ROR = 24.86; renal tubular disorder: ROR = 46.46), while voriconazole was associated with hepatobiliary disorders (ROR = 4.61) and ocular toxicity (toxic optic neuropathy: ROR = 228.80). Caspofungin demonstrated marked hepatotoxicity (cholestasis: ROR = 23.79), whereas Posaconazole and Isavuconazole showed lower mortality rates (19.56% and 22.70%, respectively). Amphotericin B demonstrated the highest mortality rate (47.14%), which was statistically significantly higher compared to other agents (χ^2^ test, p < 0.001), and life-threatening AE rates (4.97%), contrasting with Isavuconazole’s favorable safety profile (1.89% life-threatening AEs). Time-to-onset analysis revealed delayed AE onset for Isavuconazole (median: 19.5 days) versus Caspofungin (6 days).

**Conclusion:**

Significant safety variations exist among antifungal agents for IA. Amphotericin B and Caspofungin are associated with severe renal/hepatic toxicities and higher mortality, while Isavuconazole and Posaconazole may offer safer alternatives with delayed AE onset. Clinicians should prioritize drug-specific risks when tailoring treatment for IA patients.

## 1 Introduction

Invasive aspergillosis (IA), a life-threatening fungal infection caused primarily by *Aspergillus fumigatus*, poses significant clinical challenges for immunocompromised populations. Ubiquitous in environmental reservoirs including soil, decomposing vegetation, and airborne particulates, Aspergillus spores initiate infection through inhalation, subsequently germinating and invading host tissues. Hematogenous dissemination frequently results in multi-organ involvement, with pulmonary manifestations predominating, followed by neurological, renal, and sinus complications (Machado et al., 2024; Karthaus and Buchheidt, 2013). Mortality rates remain substantial despite therapeutic advances, necessitating optimized antifungal management strategies.

Current Food and Drug Administration (FDA)-approved therapies for IA encompass three principal antifungal classes: triazoles (Voriconazole, Posaconazole, Isavuconazole), polyenes (Amphotericin B and its lipid derivatives), and echinocandins (Caspofungin). While these agents demonstrate varying efficacy profiles, their safety limitations present critical clinical challenges. Voriconazole, despite its recommendation as first-line therapy, carries risks of neurotoxicity, hepatotoxicity, and complex cytochrome-mediated drug interactions. Amphotericin B formulations, though broad-spectrum, exhibit dose-limiting nephrotoxicity and infusion-related reactions. Echinocandins, while better tolerated, may induce hepatic enzyme elevations and injection-site complications (Boyer et al., 2023; Cadena et al., 2016). These safety concerns highlight the need for systematic pharmacovigilance in treatment optimization.

The FDA Adverse Event Reporting System (FAERS) serves as a vital surveillance tool for post-marketing drug safety evaluation, aggregating over 18 million spontaneous AE reports since 1968. Its utility extends beyond regulatory monitoring, enabling hypothesis generation through disproportionality analysis–a quantitative signal detection methodology that identifies disproportionate drug-AE associations relative to background reporting rates. This approach has proven particularly valuable for detecting rare or delayed-onset toxicities undetected in premarketing trials (Polep et al., 2014). Recent applications in antifungal pharmacovigilance have revealed underreported safety patterns, including azole-associated periostitis and echinocandin-induced histamine release syndromes.

This investigation employs FAERS data-mining strategies to systematically evaluate hepatotoxic, nephrotoxic, and infusion-related AEs associated with contemporary IA therapies. Through comparative analysis of AE signals across antifungal classes, we aim to: (1) quantify real-world toxicity profiles, (2) identify class-specific safety signatures, and (3) inform risk mitigation strategies for vulnerable populations. Our findings seek to complement clinical trial evidence by elucidating population-level safety patterns, ultimately guiding therapeutic decision-making in invasive fungal disease management.

## 2 Materials and methods

### 2.1 Antifungal drugs

This study focused on five antifungal agents approved by the FDA for the treatment of IA: the triazoles voriconazole (VRZ), posaconazole (PSC), and isavuconazole (ISA); the polyene amphotericin B (AMB) (including its lipid formulations); and the echinocandin caspofungin (CAS). The initial FDA approval year for each agent in the context of IA is summarized in Table 1.

**TABLE 1:** Antifungal agents approved by the FDA for IA.

U.S. FDA initial year of approval	Generic name	Brand name	Classification
1997	Amphotericin B (AmB)	Fungizone AmBisome^*^	Polyenes
2002	Voriconazole (VRZ)	Vfend	Triazoles
2006	Posaconazole (PSC)	Noxafil	Triazoles
2015	Isavuconazole (ISA)	Cresemba	Triazoles
2001	Caspofungin (CAS)	Cancidas	Echinocandins

FDA, food and drug administration; IA, invasive aspergillosis; *, Liposome dosage form.

### 2.2 Data sources and processing

**FIGURE 1:**
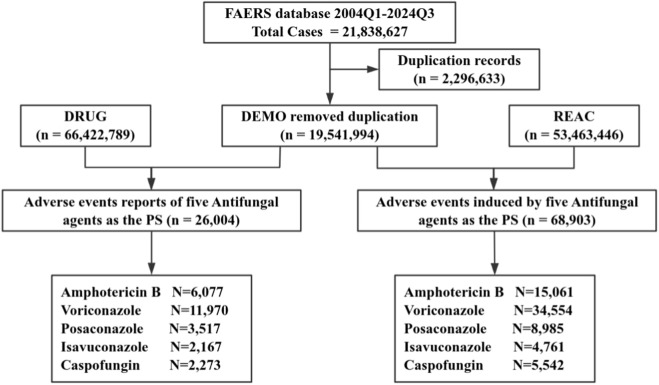
Flowchart for screening AEs related to five antifungal agents from the FAERS database.

The study analyzed 21,838,627 AE reports from the FAERS database (2004Q1–2024Q3), a global pharmacovigilance repository adhering to ICH E2B guidelines (Food and Drug Administration, 2024). The dataset comprises seven normalized tables: Demographics (DEMO), Drug Information (DRUG), Adverse Events (REAC), Patient Outcomes (OUTC), Reporting Source (RPSR), Drug Therapy Duration (THER), and Indications for Use (INDI). Figure 1 illustrates the systematic data selection workflow. To address inherent duplication from quarterly updates, we implemented a hierarchical FDA-recommended deduplication strategy by retaining reports with the latest FDA_DT for identical CASE_IDs and selecting entries with the highest PRIMARY_ID when CASE_ID-FDA_DT pairs coincided, ultimately yielding 19,541,994 unique reports (10.5% reduction) as detailed in Figure 1.

From the deduplicated dataset, 26,004 reports involving five antifungal agents were identified: AMB (n = 6,077), VRZ (n = 11,970), PSC (n = 3,517), ISA (n = 2,167), and CAS (n = 2,273). To enhance signal detection validity, only cases where these agents were designated as primary suspect (PS) drugs were included. AE terminology was standardized using MedDRA version 27.0, with Preferred Terms (PTs) systematically categorized into primary System Organ Classes (SOCs) to prevent multi-SOC duplication. This study was exempt from IRB approval as it exclusively analyzed de-identified, publicly available data from the FAERS database. All data handling procedures complied with FDA regulations for secondary analysis of pharmacovigilance data.

### 2.3 Statistical analysis

In this study, the disproportionation method was employed to evaluate the associations between five antifungal agents and AEs. Four signal calculation methods, namely the Reporting Odds Ratio (ROR), Proportional Reporting Ratio (PRR), Bayesian Confidence Propagation Neural Network (BCPNN), and Multi-item Gamma-Poisson Shrinkage (MGPS), were primarily utilized. These four signals are widely applied data mining algorithms in the analysis of the FAERS database (Sakaeda et al., 2013). The calculated ROR and PRR values, representing the strength of the association between the two drugs and AEs, both demonstrated positive correlations. To enhance the accuracy and reliability of the results, the MGPS and BCPNN methods were adopted to reduce the likelihood of false-positive outcomes. The formulas and signal detection criteria for these four algorithms are presented in Supplementary Table S1 and Table 2. Generally, a higher algorithm value indicates a more prominent signal, suggesting a stronger correlation between the drug and the occurrence of AEs.

**TABLE 2:** Four calculation methods for signal detection.

Method	Equation	Criteria
ROR	ROR = ad/bc	Lower limit of 95% CI > 1, N ≥ 3
	95% CI = e^ln(ROR)±1.96(1/a+1/b+1/c+1/d)^0.5^^	
PRR	PRR = (a(c+d))/(c(a+b))	PRR≥2, χ^2^ ≥ 4, N ≥ 3
	χ^2^ = [(ad − bc)^2^] (a + b + c + d)/[(a + b)(c + d)(a + c)(b + d)]	
BCPNN	IC = log_2_a (a+b+c+d)(a+c)(a+b)	IC_025_ > 0
	IC_025_ = e^ln(IC)-1.96(1/a+1/b+1/c+1/d)^0.5^^	
MGPS	EBGM = a (a+b+c+d)/((a+c)/(a+b))	EBGM05 > 2, N > 0
	EBGM05 = e^ln(EBGM)-1.64(1/a+1/b+1/c+1/d)^0.5^^	

a, the number of reports with suspect AEs, of the suspect drug; b, the number of reports with all other AEs, of the suspect drug; c, the number of reports with the suspect AEs, of all other drugs; d, the number of reports with all other AEs, of all other drugs; ROR, reporting odds ratio; CI, confidence interval; N, the number of co-occurrences; PRR, proportional reporting ratio; χ^2^, chi-squared; BCPNN, bayesian confidence propagation neural network; IC, information component; IC025, the lower limit of the 95% two-sided CI, of the IC; MGPS, multi-item gamma Poisson shrinker; EBGM, empirical Bayesian geometric mean; EBGM05, the lower 95% one-sided CI, of EBGM.

According to the BCPNN signal intensity criteria: 0 < IC025 < 1.5 is categorized as weak signal, 1.5 ≤ IC025 < 3.0 indicates a moderately strong signal, and IC025 ≥ 3.0 represents a strong signal. Moderately strong or strong signals must be continuously monitored within a specific time period. This study mainly assesses and contrasts moderately strong signals where IC025 ≥ 1.5.

All data cleaning and visualization operations were carried out using R Version 4.3.3, while data collation was performed with Microsoft Excel 2021.

For comparisons of categorical outcomes (e.g., mortality rates, hospitalization rates) among the five antifungal agents, Pearson’s chi-squared (χ^2^) test was employed. A two-sided p-value of less than 0.05 was considered statistically significant.

## 3 Result

### 3.1 Descriptive analysis

From January 2004 to September 2024, a total of 21,838,627 AE reports were received by the FAERS. Among them, 26,004 AE reports were related to the target antifungal agents. Specifically, there were 6,077 reports for AMB, 11,970 for VRZ, 3,517 for PSC, 2,167 for ISA, and 2,273 for CAS. In terms of gender distribution, male patients accounted for 52.99%, exceeding female patients who accounted for 34.30%. The age of patients mainly ranged from 18 to 64 years old, with a proportion of 39.99%. For individual drugs, the percentages with this 18–64 age group were as follows: 48.03% for AMB, 37.45% for VRZ, 40.60% for PSC, 25.89% for ISA, and 44.35% for CAS. These reports predominantly originated from healthcare professionals such as doctors, nurses, pharmacists, and other health professionals (e.g., nurse practitioners, clinical coordinators), constituting 69.68% of the total submissions. Additionally, consumers contributed 27.89% of the reports. Remarkably, the United States had the highest number of reported cases, totaling 9,371 cases, which accounted for 36.04% of the total. Subsequently, the United Kingdom reported 3,444 cases (13.24%), followed by France with 2,223 cases (8.55%), Japan with 2,086 cases (8.02%), and China with 1,361 cases (5.23%). Table 3 offers an in-depth analysis of the patient demographics and adverse event reports associated with the utilization of antifungal agents.

**TABLE 3:** Characteristics of AEs cases for five antifungal agents.

Characteristics	Total	AMB	VRZ	PSC	ISA	CAS
Number of reports	26,004	6,077	11,970	3,517	2,167	2,273
Gender, *n* (%)						
Female	8,919 (34.30)	2,089 (34.38)	3,935 (32.87)	1,267 (36.03)	826 (38.12)	802 (35.28)
Male	13,779 (52.99)	3,320 (54.63)	6,313 (52.74)	1,767 (50.24)	1,185 (54.68)	1,194 (52.53)
Unknown	3,306 (12.71)	668 (10.99)	1,722 (14.39)	483 (13.73)	156 (7.20)	277 (12.19)
Age (year), *n* (%)						
<18	2,376 (9.14)	750 (12.34)	953 (7.96)	354 (10.07)	65 (3.00)	254 (11.17)
18–64	10,399 (39.99)	2,919 (48.03)	4,483 (37.45)	1,428 (40.60)	561 (25.89)	1,008 (44.35)
≥65	6,766 (26.02)	1,206 (19.85)	3,714 (31.03)	822 (23.37)	475 (21.92)	549 (24.15)
Unknown	6,463 (24.85)	1,202 (19.78)	2,820 (23.56)	913 (25.96)	1,066 (49.19)	462 (20.33)
Reported person, *n* (%)						
Consumer	7,253 (27.89)	3,010 (49.53)	2,079 (17.37)	683 (19.42)	1,253 (57.82)	228 (10.03)
Health professionals	4,233 (16.28)	834 (13.72)	2247 (18.77)	774 (22.01)	83 (3.83)	295 (12.98)
Physician	7,056 (27.13)	762 (12.54)	3,760 (31.41)	1,110 (31.56)	364 (16.80)	1,060 (46.63)
Other health professional^*^	4,542 (17.47)	880 (14.48)	2,406 (20.10)	556 (15.81)	292 (13.47)	408 (17.95)
Pharmacist	2,289 (8.80)	446 (7.34)	1,121 (9.37)	345 (9.81)	161 (7.43)	216 (9.50)
Unknown	623 (2.40)	142 (2.34)	354 (2.96)	48 (1.36)	13 (0.60)	66 (2.90)
Reported countries, *n* (%)						
USA	9,371 (36.04)	1,159 (19.07)	4,493 (37.54)	1,885 (53.60)	1,469 (67.79)	365 (16.06)
Japan	2,086 (8.02)	462 (7.60)	1,234 (10.31)	92 (2.62)	13 (0.60)	285 (12.54)
France	2,223 (8.55)	227 (3.74)	1,153 (9.63)	402 (11.43)	59 (2.72)	382 (16.81)
China	1,361 (5.23)	54 (0.89)	1,035 (8.65)	122 (3.47)	40 (1.85)	110 (4.84)
UK	3,444 (13.24)	2,741 (45.10)	437 (3.65)	147 (4.18)	40 (1.85)	79 (3.48)
Outcome, *n* (%)						
Death	8,187 (31.48)	2,865 (47.14)	3,223 (26.93)	688 (19.56)	492 (22.70)	919 (40.43)
Disability	232 (0.89)	49 (0.81)	130 (1.09)	24 (0.68)	11 (0.51)	18 (0.79)
Hospitalization	4,730 (18.19)	716 (11.78)	2,482 (20.74)	704 (20.02)	338 (15.60)	490 (21.56)
Life-threatening	1,064 (4.09)	302 (4.97)	446 (3.73)	143 (4.07)	41 (1.89)	132 (5.81)
Other serious outcome	7,442 (28.62)	1651 (27.17)	4,129 (34.49)	792 (22.52)	437 (20.17)	433 (19.05)

*includes nurse practitioners, clinical research coordinators, and other certified healthcare providers.

**FIGURE 2:**
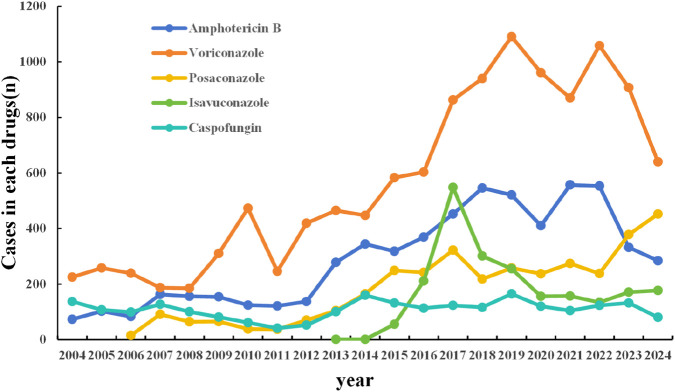
Trends of AE occurrence of five antifungal agents from 2004 to 2024.

Upon analyzing the reporting years, it was observed that for AMB and VRZ, there was an upward trajectory from 2004 to 2018, followed by a decline from 2019 onward. The number of PSC reports exhibited a steady increase over time. In contrast, the growth trend for CAS was modest. For ISA, the peak occurrence was noted in the fifth year following its market launch (as depicted in Figure 2).

### 3.2 Risk signals of system organ classes (SOCs)

**FIGURE 3:**
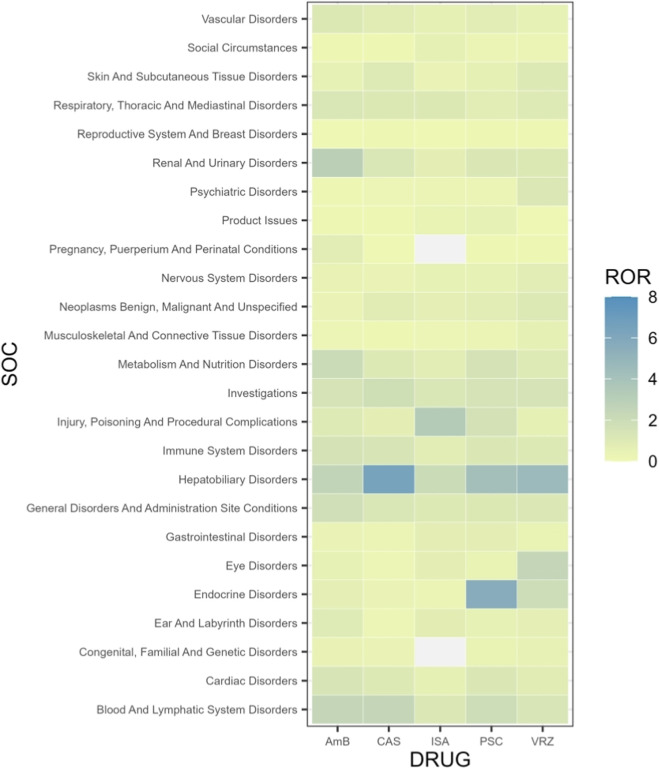
Heatmap visualization of SOCs level reporting odds ratios (RORs). Darker blue colors indicate higher RORs, whereas lighter colors represent lower RORs.

The potential AE signals were classified according to System Organ Classes (SOCs), with a total of 25 SOCs involved. The SOCs related to five antifungal agents are presented in Table 4 and Figure 3. The strongest AE signals at the SOC level for each drug were as follows. AMB: Renal and Urinary Disorders (ROR = 2.91). VRZ: Hepatobiliary Disorders (ROR = 4.61) and Eye Disorders (ROR = 2.45). PSC: Endocrine Disorders (ROR = 5.63) and Hepatobiliary Disorders (ROR = 4.18). ISA: Injury, Poisoning and Procedural Complications (ROR = 3.31). CAS: Hepatobiliary Disorders (ROR = 6.46). Detailed data for all SOCs are provided in Table 4. Notably, positive signals were detected for all five antifungal agents across three SOCs: “Hepatobiliary Disorders,” “Infections and Infestations,” and “Investigations.” However, “Infections and Infestations” was excluded from the signal analysis as it is directly related to the underlying disease diagnosis. In contrast, AEs related to “Hepatobiliary Disorders” are described, to varying degrees, in the product instructions of all five antifungal agents. This provides some validation of the positive signals detected in this study. The “Investigations” encompasses abnormal blood test results or other abnormal clinical examination findings. The presence of such findings may indicate clinically significant adverse reactions that extend beyond simple symptoms or physical signs.

**TABLE 4:** Comparison of AE risk signals at the SOC level for five antifungal agents.

SOC	AMB		VRZ		PSC		ISA		CAS	
	n	ROR (95% CI)	n	ROR (95% CI)	n	ROR (95% CI)	n	ROR (95% CI)	n	ROR (95% CI)
Blood and lymphatic system disorders	604	2.39 (2.20–2.59)^*^	788	1.34 (1.24–1.43)*	294	1.94 (1.72–2.17)*	95	1.16 (0.95–1.43)	223	2.40 (2.10–2.74)*
Cardiac disorders	560	1.40 (1.29–1.53)*	801	0.86 (0.80–0.93)	290	1.21 (1.08–1.36)*	76	0.59 (0.47–0.74)	157	1.06 (0.90–1.24)
Congenital, familial and genetic disorders	20	0.43 (0.28–0.67)	52	0.49 (0.37–0.64)	13	0.47 (0.27–0.81)	-	-	6	0.35 (0.16–0.78)
Ear and labyrinth disorders	60	0.92 (0.71–1.18)	104	0.69 (0.57–0.84)	20	0.51 (0.33–0.79)	16	0.77 (0.47–1.26)	5	0.21 (0.09–0.50)
Endocrine disorders	26	0.67 (0.46–0.99)	167	1.89 (1.63–2.20)^*^	128	5.63 (4.73–6.71)*	4	0.33 (0.12–0.87)	5	0.35 (0.15–0.85)
Eye disorders	158	0.51 (0.44–0.60)	1664	2.45 (2.34–2.58)*	84	0.46 (0.37–0.57)	63	0.65 (0.51–0.83)	22	0.19 (0.13–0.29)
Gastrointestinal disorders	535	0.39 (0.36–0.43)	1395	0.45 (0.42–0.47)	564	0.71 (0.65–0.77)	314	0.75 (0.67–0.84)	155	0.31 (0.26–0.36)
General disorders and administration site conditions	4067	1.74 (1.68–1.80)*	6770	1.14 (1.11–1.18)*	1666	1.07 (1.01–1.13)*	844	1.01 (0.94–1.09)	1170	1.26 (1.18–1.34)*
Hepatobiliary disorders	354	2.59 (2.33–2.88)*	1419	4.61 (4.38–4.87)*	336	4.18 (3.75–4.66)*	91	2.10 (1.70–2.58)*	314	6.46 (5.77–7.24)*
Immune system disorders	258	1.54 (1.36–1.74)*	389	1.01 (0.91–1.11)	120	1.20 (1.00–1.43)*	44	0.82 (0.61–1.11)	86	1.39 (1.13–1.72)*
Injury, poisoning and procedural complications	1379	0.97 (0.92–1.02)	2006	0.59 (0.57–0.62)	1276	1.59 (1.50–1.69)*	1219	3.31 (3.10–3.53)*	347	0.64 (0.58–0.72)
Investigations	1358	1.49 (1.41–1.57)*	3142	1.50 (1.45–1.56)*	804	1.47 (1.37–1.58)*	357	1.22 (1.09–1.35)*	616	1.88 (1.72–2.04)*
Metabolism and nutrition disorders	666	2.09 (1.93–2.26)*	705	0.94 (0.87–1.01)	292	1.52 (1.35–1.70)*	84	0.81 (0.65–1.01)	120	1.00 (0.83–1.20)
Musculoskeletal and connective tissue disorders	191	0.23 (0.20–0.26)	1047	0.56 (0.52–0.59)	158	0.32 (0.27–0.37)	57	0.22 (0.17–0.28)	39	0.13 (0.09–0.17)
Neoplasms benign, malignant and unspecified	152	0.37 (0.32–0.43)	966	1.05 (0.98–1.11)	198	0.82 (0.71–0.94)	89	0.69 (0.56–0.85)	131	0.88 (0.74–1.05)
Nervous system disorders	581	0.43 (0.39–0.47)	2491	0.83 (0.80–0.86)	414	0.52 (0.47–0.57)	223	0.53 (0.46–0.60)	173	0.34 (0.30–0.40)
Pregnancy, puerperium and perinatal conditions	55	0.84 (0.65–1.10)	15	0.10 (0.06–0.17)	4	0.10 (0.04–0.27)	-	-	1	0.04 (0.01–0.29)
Product issues	30	0.12 (0.09–0.18)	28	0.05 (0.03–0.07)	71	0.50 (0.39–0.63)	36	0.47 (0.34–0.66)	7	0.08 (0.04–0.17)
Psychiatric disorders	162	0.18 (0.15–0.21)	2286	1.16 (1.11–1.21)*	135	0.25 (0.21–0.30)	76	0.27 (0.21–0.33)	66	0.20 (0.15–0.25)
Renal and urinary disorders	787	2.91 (2.71–3.13)*	706	1.10 (1.02–1.19)*	196	1.18 (1.02–1.36)*	58	0.65 (0.50–0.84)	130	1.27 (1.07–1.51)*
Reproductive system and breast disorders	4	0.03 (0.01–0.08)	35	0.12 (0.09–0.17)	11	0.15 (0.08–0.26)	2	0.05 (0.01–0.20)	4	0.09 (0.03–0.23)
Respiratory, thoracic and mediastinal disorders	886	1.24 (1.15–1.32)*	1622	0.97 (0.93–1.02)	344	0.79 (0.71–0.88)	249	1.09 (0.96–1.24)	293	1.10 (0.98–1.24)
Skin and subcutaneous tissue disorders	423	0.50 (0.46–0.56)	1926	1.03 (0.98–1.08)	295	0.59 (0.53–0.67)	94	0.35 (0.29–0.43)	281	0.93 (0.83–1.05)
Social circumstances	8	0.12 (0.06–0.24)	44	0.29 (0.22–0.39)	13	0.33 (0.19–0.57)	12	0.58 (0.33–1.01)	2	0.08 (0.02–0.33)
Vascular disorders	351	1.07 (0.96–1.19)	413	0.54 (0.49–0.60)	159	0.81 (0.69–0.95)	61	0.58 (0.45–0.75)	107	0.88 (0.73–1.07)

SOC, system organ classe; N, the number of AEs, reports; ROR, reporting odds ratio; CI, confidence interval; *, positive signals detected, when N ≥ 3, lower limit of 95% CI > 1.

### 3.3 Risk signals of preferred terms (PTs)

**FIGURE 4:**
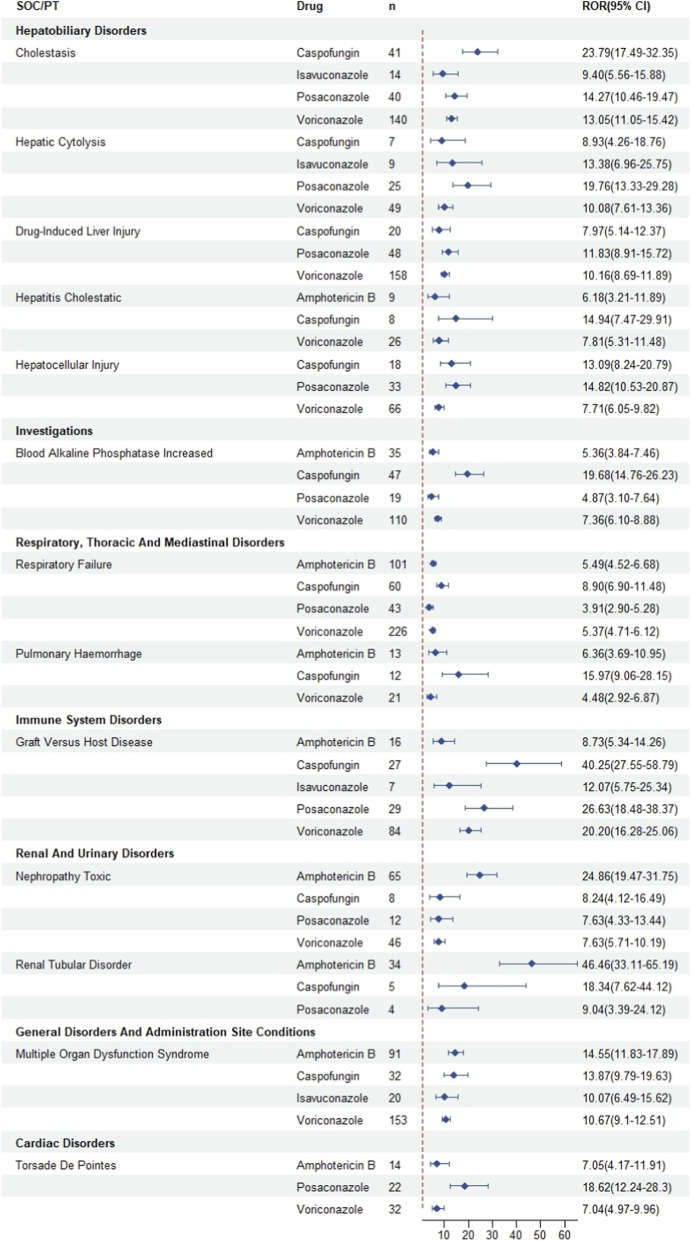
Forest plot of risk signals for antifungal agents: system organ class/preferred terms (SOC/PT) and reporting odds ratios (ROR). *N*, the number of AEs reports.

A total of 68,903 AEs associated with five antifungal agents were analyzed for risk signals. VRZ accounted for the highest proportion of reported AEs (n = 34,554, 50.1%), followed by AMB (n = 15,061, 21.9%), PSC (n = 8,985, 13.0%), CAS (n = 5,542, 8.0%), and ISA (n = 4,761, 6.9%). This disproportional AE distribution likely reflects variations in clinical utilization patterns, drug-specific pharmacokinetic properties, and intrinsic toxicity profiles. Table 5 and Figure 4 summarize the preferred terms (PTs) associated with these antifungal agents.

**TABLE 5:** Risk signals of PTs for five antifungal agents.

Drugs	SOC	PT	n	ROR (95% CI)	PRR (χ^2^)	IC (IC025)	EBGM (EBGM05)
AMB	Blood and lymphatic system disorders	Pancytopenia*	122	8.93 (7.47–10.67)	8.87 (850.00)	3.14 (2.88)	8.85 (7.62)
		Bone marrow failure*	37	6.82 (4.94–9.42)	6.81 (183.07)	2.77 (2.30)	6.80 (5.19)
		Disseminated intravascular coagulation*	31	8.49 (5.97–12.09)	8.48 (204.02)	3.08 (2.57)	8.46 (6.30)
	Cardiac disorders	Tachycardia	96	4.33 (3.54–5.29)	4.31 (243.94)	2.11 (1.81)	4.30 (3.64)
		Ventricular fibrillation*	23	8.06 (5.35–12.14)	8.05 (141.71)	3.01 (2.42)	8.03 (5.70)
		Torsade de pointes*	14	7.05 (4.17–11.91)	7.04 (72.44)	2.81 (2.07)	7.03 (4.53)
	Endocrine disorders	Diabetes insipidus	13	21.15 (12.26–36.50)	21.14 (247.92)	4.39 (3.62)	21.02 (13.32)
	Eye disorders	Ocular toxicity	4	19.07 (7.14–50.95)	19.06 (68.10)	4.25 (2.95)	18.97 (8.33)
	General disorders and administration site conditions	Chills	112	3.78 (3.14–4.55)	3.76 (226.86)	1.91 (1.64)	3.75 (3.21)
		Multiple organ dysfunction syndrome*	91	14.55 (11.83–17.89)	14.47 (1136.61)	3.85 (3.55)	14.41 (12.13)
	Hepatobiliary disorders	Hepatic function abnormal	43	4.74 (3.51–6.40)	4.73 (126.42)	2.24 (1.80)	4.73 (3.68)
		Hepatic failure*	35	4.56 (3.27–6.35)	4.55 (96.93)	2.18 (1.70)	4.55 (3.44)
		Hyperbilirubinaemia	19	7.49 (4.77–11.75)	7.48 (106.51)	2.90 (2.25)	7.47 (5.12)
	Immune system disorders	Anaphylactic shock*	26	4.19 (2.85–6.16)	4.19 (63.04)	2.06 (1.51)	4.18 (3.03)
		Graft versus host disease	16	8.73 (5.34–14.26)	8.72 (109.07)	3.12 (2.42)	8.70 (5.77)
	Investigations	Blood creatinine increased	156	9.38 (8.01–10.99)	9.29 (1152.92)	3.21 (2.98)	9.27 (8.12)
		Oxygen saturation decreased*	64	4.77 (3.73–6.10)	4.76 (189.88)	2.25 (1.89)	4.75 (3.87)
		Blood potassium decreased	62	8.14 (6.34–10.45)	8.11 (386.02)	3.02 (2.65)	8.10 (6.57)
		Alanine aminotransferase increased	60	3.82 (2.96–4.92)	3.81 (124.31)	1.93 (1.56)	3.81 (3.08)
		Aspartate aminotransferase increased	58	4.26 (3.29–5.52)	4.25 (144.13)	2.09 (1.71)	4.25 (3.42)
		Blood bilirubin increased	41	5.88 (4.33–8.00)	5.87 (165.51)	2.55 (2.11)	5.86 (4.54)
		Liver function test abnormal	40	5.19 (3.81–7.08)	5.18 (134.79)	2.37 (1.92)	5.17 (3.99)
		Blood urea increased	37	8.33 (6.03–11.51)	8.31 (237.49)	3.05 (2.58)	8.29 (6.33)
		Blood alkaline phosphatase increased	35	5.36 (3.84–7.46)	5.35 (123.51)	2.42 (1.93)	5.34 (4.04)
		Blood magnesium decreased	20	9.32 (6.01–14.46)	9.31 (147.92)	3.21 (2.58)	9.29 (6.43)
	Metabolism and nutrition disorders	Hypokalaemia	304	27.28 (24.34–30.57)	26.75 (7484.01)	4.73 (4.56)	26.56 (24.14)
		Hyperkalaemia	53	6.13 (4.68–8.02)	6.11 (226.12)	2.61 (2.21)	6.10 (4.87)
		Hypomagnesaemia	47	13.99 (10.50–18.63)	13.95 (562.72)	3.80 (3.38)	13.89 (10.93)
		Metabolic acidosis	35	4.51 (3.24–6.28)	4.50 (95.22)	2.17 (1.69)	4.50 (3.41)
	Nervous system disorders	Intracranial pressure increased*	12	8.61 (4.88–15.17)	8.60 (80.44)	3.10 (2.30)	8.58 (5.34)
	Renal and urinary disorders	Renal impairment	149	7.29 (6.20–8.57)	7.23 (799.19)	2.85 (2.61)	7.22 (6.30)
		Acute kidney injury	145	3.93 (3.34–4.63)	3.90 (313.31)	1.96 (1.72)	3.90 (3.40)
		Renal failure	123	3.52 (2.95–4.21)	3.50 (220.09)	1.81 (1.55)	3.50 (3.02)
		Nephropathy toxic	65	24.86 (19.47–31.75)	24.76 (1471.79)	4.62 (4.26)	24.59 (20.04)
		Renal disorder	47	4.00 (3.00–5.32)	3.99 (105.23)	1.99 (1.58)	3.99 (3.14)
		Renal tubular disorder*	34	46.46 (33.11–65.19)	46.36 (1489.49)	5.52 (5.02)	45.77 (34.48)
	Respiratory, thoracic and mediastinal disorders	Respiratory failure	101	5.49 (4.52–6.68)	5.46 (368.17)	2.45 (2.16)	5.46 (4.63)
		Respiratory distress*	46	6.62 (4.95–8.84)	6.60 (218.19)	2.72 (2.30)	6.59 (5.17)
		Tachypnoea*	36	10.87 (7.83–15.08)	10.84 (320.79)	3.43 (2.96)	10.81 (8.22)
		Bronchospasm	27	7.39 (5.07–10.79)	7.38 (148.66)	2.88 (2.33)	7.37 (5.37)
	Skin and subcutaneous tissue disorders	Toxic epidermal necrolysis*	30	8.06 (5.63–11.53)	8.04 (184.69)	3.01 (2.49)	8.03 (5.95)
	Vascular disorders	Shock*	44	7.90 (5.87–10.62)	7.88 (263.82)	2.98 (2.54)	7.86 (6.14)
VRZ	Blood and lymphatic system disorders	Hypoprothrombinaemia*	5	27.98 (11.55–67.76)	27.98 (127.74)	4.78 (3.59)	27.49 (13.12)
	Cardiac disorders	Torsade de pointes	32	7.04 (4.97–9.96)	7.03 (164.82)	2.81 (2.30)	7.00 (5.24)
		Cardiovascular insufficiency*	13	13.34 (7.73–23.03)	13.34 (147.07)	3.73 (2.95)	13.23 (8.38)
	Endocrine disorders	Inappropriate antidiuretic hormone secretion*	61	10.88 (8.45–14.00)	10.86 (542.33)	3.43 (3.06)	10.79 (8.74)
		Adrenal insufficiency	32	5.13 (3.62–7.25)	5.12 (105.84)	2.35 (1.85)	5.11 (3.82)
		Cushing's syndrome*	20	8.59 (5.53–13.33)	8.58 (133.28)	3.09 (2.46)	8.54 (5.91)
	Eye disorders	Visual impairment	318	4.67 (4.18–5.22)	4.64 (907.26)	2.21 (2.05)	4.63 (4.22)
		Photophobia	106	10.17 (8.40–12.31)	10.14 (867.99)	3.33 (3.05)	10.08 (8.59)
		Toxic optic neuropathy*	49	228.80 (169.46–308.93)	228.48 (9662.84)	7.64 (7.20)	199.07 (154.84)
		Photopsia*	43	11.83 (8.76–15.97)	11.81 (422.40)	3.55 (3.12)	11.73 (9.12)
		Halo vision*	38	59.55 (43.06–82.35)	59.48 (2103.69)	5.84 (5.37)	57.31 (43.69)
		Xanthopsia*	38	207.57 (147.91–291.30)	207.34 (6876.69)	7.51 (7.02)	182.84 (137.70)
		Dyschromatopsia	37	87.28 (62.66–121.57)	87.19 (2983.31)	6.37 (5.89)	82.56 (62.57)
		Visual field defect	33	7.73 (5.49–10.88)	7.72 (192.15)	2.94 (2.45)	7.69 (5.77)
		Chromatopsia	31	70.00 (48.84–100.35)	69.94 (2015.07)	6.06 (5.54)	66.94 (49.53)
		Chloropsia*	19	336.23 (204.66–552.38)	336.04 (5209.22)	8.11 (7.41)	275.99 (182.17)
	Gastrointestinal disorders	Cheilitis	41	13.03 (9.58–17.73)	13.02 (451.13)	3.69 (3.24)	12.92 (9.99)
		Lip blister*	17	10.74 (6.66–17.30)	10.73 (149.03)	3.41 (2.73)	10.67 (7.16)
	General disorders and administration site conditions	Multiple organ dysfunction syndrome	153	10.67 (9.10–12.51)	10.63 (1325.83)	3.40 (3.17)	10.56 (9.24)
		Necrosis*	25	7.65 (5.17–11.34)	7.65 (143.76)	2.93 (2.36)	7.61 (5.48)
	Hepatobiliary disorders	Hepatic function abnormal	203	9.83 (8.56–11.29)	9.78 (1590.93)	3.28 (3.08)	9.72 (8.66)
		Hepatotoxicity	188	15.31 (13.26–17.68)	15.23 (2476.28)	3.92 (3.70)	15.09 (13.38)
		Drug-induced liver injury	158	10.16 (8.69–11.89)	10.12 (1290.64)	3.33 (3.10)	10.06 (8.82)
		Cholestasis	140	13.05 (11.05–15.42)	13.00 (1538.88)	3.69 (3.45)	12.90 (11.22)
		Hepatocellular injury*	66	7.71 (6.05–9.82)	7.70 (382.73)	2.94 (2.58)	7.66 (6.26)
		Liver injury	63	5.24 (4.09–6.72)	5.24 (215.28)	2.38 (2.02)	5.22 (4.25)
		Hepatic cytolysis*	49	10.08 (7.61–13.36)	10.07 (397.77)	3.32 (2.91)	10.01 (7.91)
		Hyperbilirubinaemia	38	6.54 (4.76–9.00)	6.53 (177.41)	2.70 (2.24)	6.51 (4.99)
		Hepatitis cholestatic	26	7.81 (5.31–11.48)	7.80 (153.41)	2.96 (2.40)	7.77 (5.63)
		Hypertransaminasaemia	26	8.07 (5.49–11.86)	8.06 (159.99)	3.00 (2.45)	8.02 (5.81)
	Immune system disorders	Graft versus host disease	84	20.20 (16.28–25.06)	20.15 (1509.58)	4.32 (4.00)	19.91 (16.62)
		Hypogammaglobulinaemia*	15	4.82 (2.90–8.00)	4.82 (45.24)	2.26 (1.54)	4.81 (3.14)
	Investigations	Alanine aminotransferase increased	131	3.64 (3.06–4.32)	3.63 (249.11)	1.86 (1.61)	3.62 (3.14)
		Aspartate aminotransferase increased	119	3.81 (3.19–4.57)	3.80 (245.64)	1.93 (1.66)	3.80 (3.27)
		Blood alkaline phosphatase increased*	110	7.36 (6.10–8.88)	7.34 (600.22)	2.87 (2.60)	7.31 (6.25)
		Liver function test abnormal	108	6.12 (5.07–7.40)	6.11 (459.86)	2.61 (2.33)	6.09 (5.20)
		Gamma-glutamyltransferase increased	100	7.52 (6.18–9.15)	7.50 (560.76)	2.90 (2.61)	7.47 (6.33)
		Electrocardiogram qt prolonged	83	4.06 (3.27–5.04)	4.05 (190.48)	2.02 (1.70)	4.04 (3.38)
		Blood bilirubin increased	68	4.25 (3.35–5.40)	4.25 (168.45)	2.08 (1.74)	4.24 (3.47)
		Transaminases increased	57	4.44 (3.42–5.76)	4.43 (151.16)	2.15 (1.77)	4.42 (3.56)
		Fluoride increased*	32	2593.93 (1470.22–4576.49)	2591.52 (30,871.23)	9.92 (9.27)	966.10 (600.76)
	Musculoskeletal and connective tissue disorders	Periostitis*	168	1231.12 (1005.00–1508.12)	1225.14 (114,401.60)	9.41 (9.15)	682.51 (575.92)
		Rhabdomyolysis*	94	3.95 (3.23–4.84)	3.94 (206.00)	1.98 (1.68)	3.93 (3.32)
		Hypertrophic osteoarthropathy*	20	669.39 (395.95–1131.67)	669.01 (9297.71)	8.87 (8.14)	466.58 (300.69)
	Nervous system disorders	Neurotoxicity	120	12.86 (10.75–15.40)	12.82 (1297.62)	3.67 (3.41)	12.72 (10.95)
		Altered state of consciousness*	106	8.92 (7.37–10.80)	8.90 (739.00)	3.15 (2.87)	8.85 (7.54)
		Posterior reversible encephalopathy syndrome*	28	5.42 (3.74–7.86)	5.42 (100.61)	2.43 (1.90)	5.41 (3.96)
	Psychiatric disorders	Hallucination	448	10.75 (9.79–11.80)	10.62 (3882.46)	3.40 (3.26)	10.56 (9.76)
		Hallucination, visual	315	27.67 (24.74–30.94)	27.42 (7882.35)	4.75 (4.59)	26.96 (24.55)
		Delirium	142	7.35 (6.23–8.67)	7.33 (772.57)	2.87 (2.62)	7.30 (6.35)
		Mental disorder	88	3.50 (2.84–4.32)	3.50 (156.51)	1.80 (1.50)	3.49 (2.93)
		Disorganised speech	57	64.79 (49.69–84.46)	64.68 (3429.61)	5.96 (5.57)	62.11 (49.75)
		Hallucination, auditory	45	4.91 (3.66–6.58)	4.90 (139.41)	2.29 (1.86)	4.89 (3.83)
		Hallucinations, mixed	29	12.26 (8.51–17.67)	12.25 (297.34)	3.60 (3.08)	12.16 (8.96)
	Renal and urinary disorders	Nephropathy toxic*	46	7.63 (5.71–10.19)	7.62 (263.22)	2.92 (2.50)	7.59 (5.95)
	Respiratory, thoracic and mediastinal disorders	Respiratory failure	226	5.37 (4.71–6.12)	5.34 (794.67)	2.41 (2.22)	5.32 (4.77)
		Haemoptysis	91	5.60 (4.55–6.88)	5.59 (341.48)	2.48 (2.18)	5.57 (4.69)
		Pulmonary haemorrhage*	21	4.48 (2.92–6.87)	4.48 (56.52)	2.16 (1.54)	4.47 (3.12)
	Skin and subcutaneous tissue disorders	Photosensitivity reaction	397	43.06 (38.96–47.61)	42.58 (15,690.35)	5.37 (5.23)	41.46 (38.12)
		Actinic keratosis*	110	72.90 (60.20–88.29)	72.67 (7425.36)	6.12 (5.84)	69.44 (59.16)
		Dermatitis bullous*	36	8.78 (6.33–12.19)	8.78 (246.66)	3.13 (2.65)	8.73 (6.64)
		Pseudoporphyria*	24	124.43 (82.08–188.63)	124.34 (2716.85)	6.85 (6.25)	115.12 (81.27)
		Lentigo*	20	69.35 (44.30–108.56)	69.31 (1288.49)	6.05 (5.41)	66.37 (45.61)
PSC	Blood and lymphatic system disorders	Febrile neutropenia	60	6.20 (4.81–7.99)	6.16 (259.61)	2.62 (2.25)	6.16 (4.98)
	Cardiac disorders	Torsade de pointes*	22	18.62 (12.24–28.30)	18.57 (364.68)	4.21 (3.61)	18.52 (13.04)
		Pericardial effusion*	18	5.33 (3.36–8.47)	5.33 (63.20)	2.41 (1.75)	5.32 (3.61)
	Endocrine disorders	Pseudoaldosteronism	61	3335.87 (2436.70–4566.84)	3313.23 (129,508.39)	11.05 (10.62)	2124.72 (1633.68)
	Hepatobiliary disorders	Drug-induced liver injury*	48	11.83 (8.91–15.72)	11.77 (472.55)	3.56 (3.14)	11.75 (9.27)
		Cholestasis*	40	14.27 (10.46–19.47)	14.21 (490.20)	3.83 (3.37)	14.18 (10.93)
		Hepatotoxicity	36	11.17 (8.05–15.51)	11.13 (331.52)	3.47 (3.00)	11.11 (8.45)
		Hepatocellular injury*	33	14.82 (10.53–20.87)	14.77 (422.68)	3.88 (3.38)	14.74 (11.07)
		Hepatic cytolysis*	25	19.76 (13.33–29.28)	19.71 (442.50)	4.30 (3.73)	19.64 (14.14)
	Immune system disorders	Graft versus host disease*	29	26.63 (18.48–38.37)	26.54 (709.79)	4.72 (4.20)	26.43 (19.47)
	Investigations	Electrocardiogram qt prolonged	48	9.05 (6.81–12.02)	9.01 (341.40)	3.17 (2.76)	9.00 (7.09)
		Liver function test abnormal	28	6.09 (4.20–8.83)	6.08 (118.67)	2.60 (2.06)	6.07 (4.45)
	Metabolism and nutrition disorders	Hypokalaemia	91	13.48 (10.96–16.57)	13.35 (1038.33)	3.74 (3.43)	13.32 (11.21)
		Hypomagnesaemia	21	10.45 (6.80–16.03)	10.42 (178.63)	3.38 (2.76)	10.41 (7.27)
		Hypophosphataemia*	14	13.01 (7.70–21.99)	12.99 (154.66)	3.70 (2.95)	12.97 (8.36)
	Nervous system disorders	Neurotoxicity*	17	6.95 (4.32–11.19)	6.94 (86.35)	2.79 (2.11)	6.93 (4.65)
	Renal and urinary disorders	Nephropathy toxic*	12	7.63 (4.33–13.44)	7.62 (68.92)	2.93 (2.13)	7.61 (4.74)
	Respiratory, thoracic and mediastinal disorders	Respiratory failure*	43	3.91 (2.90–5.28)	3.90 (92.61)	1.96 (1.53)	3.89 (3.03)
		Pulmonary Mass*	14	6.40 (3.79–10.82)	6.39 (63.67)	2.68 (1.93)	6.39 (4.12)
ISA	Gastrointestinal disorders	Dysphagia*	31	4.19 (2.94–5.96)	4.17 (74.69)	2.06 (1.55)	4.17 (3.10)
	General disorders and administration site conditions	Death*	266	4.12 (3.64–4.67)	3.95 (593.93)	1.98 (1.80)	3.95 (3.56)
		Multiple organ dysfunction syndrome*	20	10.07 (6.49–15.62)	10.03 (162.48)	3.32 (2.69)	10.02 (6.94)
	Hepatobiliary disorders	Cholestasis*	14	9.40 (5.56–15.88)	9.37 (104.65)	3.23 (2.48)	9.37 (6.04)
		Hepatotoxicity	14	8.18 (4.84–13.83)	8.16 (87.95)	3.03 (2.28)	8.16 (5.26)
	Respiratory, thoracic and mediastinal disorders	Haemoptysis*	13	5.79 (3.36–9.98)	5.78 (51.34)	2.53 (1.76)	5.77 (3.66)
CAS	Blood and lymphatic system disorders	Pancytopenia*	29	5.74 (3.99–8.27)	5.72 (112.91)	2.51 (1.99)	5.71 (4.21)
		Eosinophilia*	26	16.54 (11.25–24.33)	16.47 (377.27)	4.04 (3.48)	16.44 (11.91)
		Agranulocytosis	20	12.26 (7.90–19.02)	12.22 (205.81)	3.61 (2.98)	12.20 (8.45)
		Disseminated intravascular coagulation*	20	14.90 (9.60–23.13)	14.85 (258.08)	3.89 (3.26)	14.83 (10.27)
	General disorders and administration site conditions	Multiple organ dysfunction syndrome*	32	13.87 (9.79–19.63)	13.79 (379.25)	3.78 (3.28)	13.77 (10.30)
	Hepatobiliary disorders	Cholestasis*	41	23.79 (17.49–32.35)	23.62 (886.15)	4.56 (4.11)	23.56 (18.21)
		Hepatic function abnormal	31	9.31 (6.54–13.26)	9.27 (228.54)	3.21 (2.70)	9.26 (6.89)
		Hepatic failure	27	9.58 (6.56–13.99)	9.54 (206.35)	3.25 (2.71)	9.53 (6.95)
		Liver disorder	21	5.21 (3.39–8.00)	5.19 (71.10)	2.38 (1.76)	5.19 (3.63)
		Drug-induced liver injury	20	7.97 (5.14–12.37)	7.95 (121.38)	2.99 (2.36)	7.94 (5.50)
		Hepatotoxicity	19	9.55 (6.08–14.98)	9.52 (144.75)	3.25 (2.60)	9.51 (6.52)
		Hepatocellular injury*	18	13.09 (8.24–20.79)	13.05 (200.01)	3.70 (3.04)	13.03 (8.85)
	Immune system disorders	Graft versus host disease*	27	40.25 (27.55–58.79)	40.06 (1024.09)	5.32 (4.77)	39.90 (29.06)
		Haemophagocytic lymphohistiocytosis*	7	13.13 (6.25–27.57)	13.11 (78.23)	3.71 (2.69)	13.10 (7.04)
	Investigations	Blood alkaline phosphatase increased	47	19.68 (14.76–26.23)	19.52 (824.63)	4.28 (3.87)	19.48 (15.32)
		Alanine aminotransferase increased	46	7.99 (5.98–10.69)	7.94 (278.93)	2.99 (2.56)	7.93 (6.22)
		Aspartate aminotransferase increased	42	8.42 (6.21–11.41)	8.36 (272.30)	3.06 (2.62)	8.36 (6.48)
		Gamma-glutamyltransferase increased	31	14.53 (10.21–20.69)	14.45 (387.79)	3.85 (3.34)	14.43 (10.74)
		Blood lactate dehydrogenase increased*	28	18.02 (12.43–26.13)	17.93 (446.96)	4.16 (3.62)	17.90 (13.12)
		Blood bilirubin increased	27	10.55 (7.23–15.40)	10.50 (232.02)	3.39 (2.84)	10.49 (7.65)
CAS	Metabolism and nutrition disorders	Hypokalaemia	28	6.68 (4.61–9.68)	6.65 (134.44)	2.73 (2.20)	6.65 (4.87)
	Nervous system disorders	Encephalopathy*	16	7.15 (4.38–11.69)	7.14 (84.38)	2.83 (2.13)	7.13 (4.73)
	Respiratory, thoracic and mediastinal disorders	Respiratory failure*	60	8.90 (6.90–11.48)	8.82 (415.90)	3.14 (2.77)	8.81 (7.12)
		Acute respiratory distress syndrome	24	14.68 (9.83–21.92)	14.62 (304.10)	3.87 (3.29)	14.60 (10.43)
	Skin and subcutaneous tissue disorders	Drug reaction with eosinophilia and systemic symptoms*	40	17.99 (13.18–24.56)	17.87 (635.99)	4.16 (3.70)	17.84 (13.75)
		Rash maculo-papular	22	11.02 (7.25–16.76)	10.98 (199.51)	3.46 (2.85)	10.97 (7.73)

*AEs, not listed in the respective drug labels; PT, preferred term; N, the number of AEs, reports; ROR, reporting odds ratio; CI, confidence interval; PRR, proportional reporting ratio, χ^2^, chi-squared; IC, information component; IC, 025, the lower limit of 95% CI, of the IC, EBGM, empirical Bayesian geometric mean; EBGM, 05, the lower limit of 95% CI, of EBGM.

The most notable PT-level signals for each drug were: AMB: renal tubular disorder (ROR = 46.46) and nephropathy toxic (ROR = 24.86). VRZ: toxic optic neuropathy (ROR = 228.80) and actinic keratosis (ROR = 72.90). PSC: pseudoaldosteronism (ROR = 3335.87) and torsade de pointes (ROR = 18.62). ISA: death (ROR = 4.12). CAS: cholestasis (ROR = 23.79). A substantial proportion of these strong signals, marked with an asterisk (*) in Table 5, were not listed in the official drug labels, highlighting potential underrecognized risks. Full details are provided in Table 5 and Figure 4.

### 3.4 Time-to-onset (TTO) of AEs induced by five antifungal agents

**FIGURE 5:**
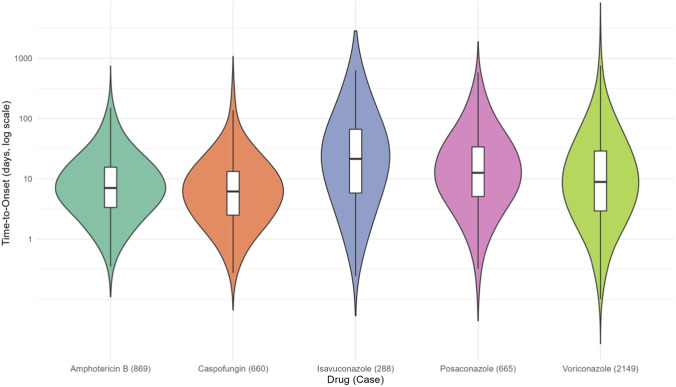
Comparative violin plot analysis of time-to-onset distributions for AEs induced by five antifungal agents.

The time-to-onset (TTO) profiles of AEs varied significantly among the five antifungal agents, as visualized in the violin plot (Figure 5). ISA exhibited the longest median TTO (19.5 days, IQR: 5–63.25 days), followed by PSC (13 days, IQR: 5–37 days) and VRZ (9 days, IQR: 3–30 days). In contrast, CAS and AMB showed shorter median TTO values (6 days and 7 days, respectively), with narrower interquartile ranges (IQR: 3–16 days and 3–15 days). Notably, the mean TTO values were substantially higher than the medians for all drugs (e.g., 55.9 days for ISA vs. 19.5 days median), reflecting skewed distributions with extreme outliers, particularly for VRZ (maximum TTO: 4,250 days) and AMB (maximum TTO: 3,964 days). The simulated violin plots (log-scale) further highlighted the right-skewed distributions and variability in TTO patterns, with CAS demonstrating the most concentrated distribution. These findings suggest distinct safety profiles among the drugs, where ISA and PSC are associated with delayed AE onset, while CAS and AMB may provoke earlier reactions. The temporal relationship between drug initiation and AE onset, as evidenced by the TTO analysis, supports a potential drug-related etiology for many of the reported events.

### 3.5 Comparison of AE outcomes among five antifungal agents

**FIGURE 6:**
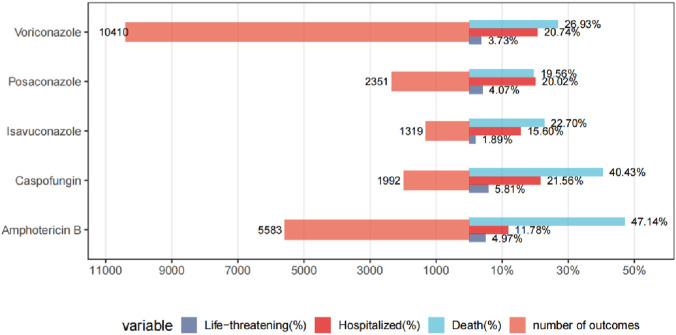
Comparative AE outcomes of five antifungal agents.

As shown in Figure 6, analysis of AE outcomes from the FAERS database revealed distinct safety profiles among AMB, VRZ, PSC, ISA, and CAS. AMB demonstrated the highest mortality rate (47.14%), followed by CAS (40.43%) and VRZ (26.93%), whereas PSC and ISA exhibited markedly lower mortality rates (19.56% and 22.70%, respectively). ISA was associated with the lowest incidence of life-threatening AEs (1.89%), contrasting sharply with CAS (5.81%) and AMB (4.97%). Hospitalization rates ranged from 15.60% (ISA) to 21.56% (CAS), with VRZ and PSC showing intermediate values (20.74% and 20.02%, respectively). Notably, VRZ, despite having the highest number of reported outcomes (n = 10,410), displayed moderate severity across metrics, while AMB, with fewer cases (n = 5,583), was linked to disproportionately severe outcomes. These findings underscore the divergent safety profiles of the evaluated drugs, with ISA and PSC emerging as favorable options in terms of mortality and morbidity, whereas AMB and CAS posed elevated risks for severe AEs.

## 4 Discussion

### 4.1 Key findings and mechanistic insights

This pharmacovigilance study documents substantial heterogeneity in the safety profiles of five FDA-approved antifungal agents for invasive aspergillosis (IA). AMB exhibited the highest mortality (47.14%) and life-threatening AE rates (4.97%), driven by profound renal toxicity (e.g., nephropathy toxic: ROR = 24.86), while CAS also posed significant risks (40.43%), underscoring the need for cautious use in critically ill patients. At the SOC level, CAS showed moderate hepatobiliary risks (ROR = 6.46), but its cholestasis signal at the PT level was markedly higher (ROR = 23.79), suggesting specific hepatic complications. VRZ demonstrated strong hepatobiliary (ROR = 4.61) and ocular toxicity signals (toxic optic neuropathy: ROR = 228.80). In contrast, ISA and PSC showed lower mortality (22.70% and 19.56%, respectively) and delayed AE onset (median TTO: 19.5 and 13 days). These findings underscore the need for agent-specific risk stratification, such as prioritizing ISA in patients with renal impairment and avoiding VRZ in those with pre-existing hepatic dysfunction.

The divergent safety outcomes likely stem from differences in pharmacological mechanisms and toxicity pathways. The pronounced nephrotoxicity associated with AMB can be attributed to its cationic polyene structure, which preferentially binds to renal tubular cell membrane cholesterol. This interaction disrupts membrane integrity, leading to increased permeability and subsequent apoptotic cell death (Tiphine et al., 1999; Sabra and Branch, 1990), consistent with its high renal tubular disorder signals (ROR = 46.46). VRZ’s hepatic metabolism via cytochrome P450 isoforms may explain its hepatobiliary risks (Dolton and McLachlan, 2014), while its retinal affinity aligns with ocular toxicity. ISA’s delayed TTO and favorable profile may relate to its balanced tissue distribution and lower drug-drug interaction potential (Ellsworth and Ostrosky-Zeichner, 2020). CAS’s hepatic signals could reflect off-target effects on hepatic transporters or immune-mediated cholestasis (Kartsonis et al., 2003). These mechanistic inferences align with preclinical studies but warrant further pharmacokinetic validation.

### 4.2 Comparison with existing literature and clinical implications

Our results corroborate prior real-world analyses. AMB’s nephrotoxicity and high mortality mirror findings from cohort studies (Cadena et al., 2016), while VRZ’s hepatotoxicity and visual disturbances are well-documented in clinical trials (Boyer et al., 2023). Notably, ISA’s lower AE severity aligns with the SECURE trial, which reported fewer hepatobiliary events compared to VRZ (Maertens et al., 2016). However, CAS’s hepatotoxicity contrasts with its perceived safety in guidelines, suggesting underrecognized risks in post-marketing settings. Discrepancies may arise from differences in study design (e.g., spontaneous reporting vs. controlled trials) or population characteristics, emphasizing the complementary role of pharmacovigilance data.

Clinically, these findings advocate for personalized antifungal selection. ISA and PSC may be prioritized in patients with renal impairment or high comorbidity burdens, whereas VRZ requires vigilant hepatic and ocular monitoring. AMB should be reserved for refractory cases due to its toxicity burden. Regulatory authorities should consider label updates for CAS to reflect hepatotoxicity risks, and clinicians should remain alert to unlabeled AEs, such as AMB-associated disseminated intravascular coagulation (DIC; ROR = 8.49) and CAS-linked hemophagocytic lymphohistiocytosis (HLH; ROR = 13.13).

These signals, though not listed in official prescribing information, carry significant clinical implications. For instance, DIC, a life-threatening coagulopathy, may arise from AMB’s direct endothelial toxicity or immune-mediated platelet activation (Cutaia et al., 1993). Similarly, CAS-induced HLH—a hyperinflammatory syndrome—could stem from dysregulated immune responses triggered by β-glucan exposure (Walker and Munro, 2020; Wagner et al., 2023). These unlabeled AEs require close clinical monitoring. Patients receiving AMB should undergo routine coagulation monitoring (e.g., D-dimer, platelet counts), particularly in prolonged therapy or high-dose regimens. For CAS, unexplained cytopenias or febrile syndromes should prompt evaluation for HLH, including ferritin and soluble IL-2 receptor levels. Such proactive measures could mitigate severe outcomes, as delayed recognition of these AEs may exacerbate morbidity or mortality. Regulatory agencies should prioritize reviewing these signals to update drug labels, ensuring clinicians are informed of potential risks.

### 4.3 Limitations and future directions

This study has several limitations inherent to the analysis of spontaneous reporting system data. First, reports in the FAERS database are subject to reporting biases, under-reporting, and confounding by indication or concomitant medications. Second, disproportionality analyses identify statistical associations but cannot establish causality. Therefore, the observed adverse outcomes may not be directly attributable solely to the antifungal agents, as confounding by the underlying severe illness or concomitant medications cannot be ruled out. Third, the FAERS data contain inconsistent or missing dosage information, precluding a robust assessment of dose-response relationships, which is crucial for understanding toxicity thresholds. Fourth, due to the spontaneous reporting nature, data on the persistence or reversibility of AEs following drug withdrawal are scarce and unsystematic, limiting insights into long-term safety outcomes.

Future studies should prioritize prospective cohorts with detailed dosing, therapeutic drug monitoring, and structured follow-up data to validate these safety signals and establish causality. Additionally, pharmacokinetic and pharmacodynamic studies are warranted to elucidate the mechanisms behind unlabeled AEs (e.g., CAS-associated hemophagocytic lymphohistiocytosis). Moreover, integrating real-world evidence from electronic health records, which provide more comprehensive patient context and treatment courses, could complement spontaneous reporting data and yield a more holistic safety profile.

## 5 Conclusion

In summary, this large-scale pharmacovigilance analysis highlights critical safety variations among IA antifungals, with AMB and CAS posing higher risks of severe organ toxicity and mortality. ISA and PSC emerge as safer alternatives, particularly in vulnerable populations. These findings reinforce the importance of post-marketing surveillance and risk-adapted treatment strategies to optimize IA management. Prospective studies focusing on unlabeled AEs (e.g., CAS-associated hemophagocytic lymphohistiocytosis) are needed to validate causality and inform guideline updates.

## Data Availability

The original contributions presented in the study are included in the article/Supplementary Material, further inquiries can be directed to the corresponding author.
